# Extracellular Vesicles and MicroRNA: Putative Role in Diagnosis and Treatment of Diabetic Retinopathy

**DOI:** 10.3390/antiox9080705

**Published:** 2020-08-04

**Authors:** Beatriz Martins, Madania Amorim, Flávio Reis, António Francisco Ambrósio, Rosa Fernandes

**Affiliations:** 1 Coimbra Institute for Clinical and Biomedical Research (iCBR), Faculty of Medicine, University of Coimbra, 3000-548 Coimbra, Portugal; mrro_54@hotmail.com (B.M.); madaniyah.gaffur@gmail.com (M.A.); freis@fmed.uc.pt (F.R.); afambrosio@fmed.uc.pt (A.F.A.); 2 Center for Innovative Biomedicine and Biotechnology, University of Coimbra, 3000-548 Coimbra, Portugal; 3 Association for Innovation and Biomedical Research on Light and Image (AIBILI), 3000-548 Coimbra, Portugal

**Keywords:** diabetic retinopathy (DR), inflammation, oxidative stress, angiogenesis, extracellular vesicles, miRNA, biomarkers, antioxidants

## Abstract

Diabetic retinopathy (DR) is a complex, progressive, and heterogenous retinal degenerative disease associated with diabetes duration. It is characterized by glial, neural, and microvascular dysfunction, being the blood-retinal barrier (BRB) breakdown a hallmark of the early stages. In advanced stages, there is formation of new blood vessels, which are fragile and prone to leaking. This disease, if left untreated, may result in severe vision loss and eventually legal blindness. Although there are some available treatment options for DR, most of them are targeted to the advanced stages of the disease, have some adverse effects, and many patients do not adequately respond to the treatment, which demands further research. Oxidative stress and low-grade inflammation are closely associated processes that play a critical role in the development of DR. Retinal cells communicate with each other or with another one, using cell junctions, adhesion contacts, and secreted soluble factors that can act in neighboring or long-distance cells. Another mechanism of cell communication is via secreted extracellular vesicles (EVs), through exchange of material. Here, we review the current knowledge on deregulation of cell-to-cell communication through EVs, discussing the changes in miRNA expression profiling in body fluids and their role in the development of DR. Thereafter, current and promising therapeutic agents for preventing the progression of DR will be discussed.

## 1. Introduction

Diabetes mellitus (DM) is one of the most common metabolic disorders that has become both a major public health threat and an economic burden for society. According to estimates from the International Diabetes Federation (IDF) in 2019, 463 million adults (one in eleven between 20–79 years) were living with diabetes and this number is expected to increase to around 700 million by 2045 [1]. Diabetes is a systemic disease that can affect almost any part of the body, including the eye, especially the retinal tissue, causing a microvascular complication known as diabetic retinopathy (DR). Diabetic retinopathy is a leading cause of visual impairment among working-age adults worldwide [2], affecting more than 149 million individuals [3]. The global prevalence of this disease has increased to epidemic proportions [4], and is responsible for 50,000 new cases of retinal neovascularization and diabetic macular edema worldwide every year [5]. This means that it affects around one-third of the diabetic population, with approximately one-tenth of these patients having vision-threatening retinopathy [6,7]. Although DR is not a mortal illness, it leads to emotional distress and reduces daily life functionality, thus significantly impacting the quality of life in patients with advanced stages of the disease. In addition to the visual impairment, it is associated with significant economic consequences for public health systems [7].

During the first two decades after the onset of diabetes, almost all type 1 diabetes (T1D) cases develop DR and about two thirds of patients with type 2 diabetes (T2D) also have some form of the disease [8]. DR is a silent complication that in its early stages causes no symptoms. However, chronic hyperglycemia can produce noticeable retina damage over time, involving blood and other fluids leakage out of retinal capillaries, which may result in cloudy or blurred vision. Therefore, if left untreated DR may result in severe visual impairment or even blindness. Although over the past several decades significant advances have been made and a variety of treatments for DR are currently available, none of them are yet curative. In the early stages of the disease, a tight blood glucose control and regular monitoring can help prevent its progression to more advanced stages. In advanced stages, the main treatments of DR include intraocular injections of anti-VEGF antibodies, laser treatments, and vitrectomy [7,9]. Although the results are encouraging and these treatments can halt the progression of DR or even temporarily improve the vision loss, they are associated with two major concerns/limitations: Can produce several adverse effects and are ineffective in some of the patients (non-responders). A better understanding of the exact cellular and molecular mechanisms underlying DR and the discovery of reliable early biomarkers will decisively contribute to the identification of putative targets for the development of new and more effective options than existing treatments offer.

Extracellular vesicles (EVs) are a heterogenous population of membranous vesicles that can be released into the extracellular milieu by the majority of the cells of the body [10]. They can be classified into three categories (microvesicles, exosomes, and apoptotic bodies) based on their size and mode of biogenesis. It is now known that tissue homeostasis strongly depends on an effective cell-to-cell communication mediated by EVs. These vesicles released from the parent cells are extracellular carriers of both proteins, RNA, and miRNA, which are capable of inducing a wide range of effects on recipient cells [11]. In fact, many miRNAs were described to play critical roles in the regulation of several cellular processes, including cell cycle, proliferation, and apoptosis [12], contributing therefore to the maintenance of the retinal tissue homeostasis. Hence, it is expected that miRNA expression dysregulation also affects those processes, directly contributing to pathological conditions, such as DR. Importantly, changes in the miRNA expression profile may reflect the pathological state, highlighting the putative use of miRNAs as biomarkers to predict DR progression [13].

Herein, we describe and discuss how inflammation and oxidative stress contribute to retinal endothelial cells dysfunction in DR, highlighting the deregulation of cell-to-cell communication mediated by extracellular vesicles. Moreover, we will summarize the most recent studies about the role of miRNAs in the pathogenesis of DR and the potential beneficial effects of therapeutic agents with anti-inflammatory and antioxidant properties.

## 2. Pathophysiologic Changes and Clinical Definition of DR

Diabetic retinopathy is a progressive disease, whose major risk factor is the duration of diabetes. It develops through a series of stages of increasing degrees of severity [14] and affects the microvascular circulation of the retina, compromising the integrity and function of retinal tissue. Endothelial cells, the building blocks of the microvasculature, are particularly sensitive to sustained hyperglycemia-induced damage [15,16]. The retina is not only a network of blood vessels, rather it is a multilayered neuronal (photoreceptors, and horizontal, bipolar, amacrine and ganglion cells) and glial (astrocytes, Müller cells, and microglia) cells that covers approximately 95% of the tissue, with blood vessels (endothelial cells and pericytes) comprising only a small portion (<5%) [17]. In healthy conditions, a synergistic interaction between retinal neurons, glial cells, and blood vessels contributes to the autoregulation of vascular flow and metabolic activity. Indeed, retinal blood vessels provide nutrients to the neural tissue, and neuroglial cells are essential players in transmission of visual information, being therefore crucial to the maintenance of the retinal homeostasis [18]. Thus, the intimate relationship between neuroglial and vascular networks is only possible by a metabolic synergy and cell-to-cell communication [18]. Although microvascular changes are found in DR and its diagnosis is based on their screening, many studies using electrophysiological tests pointed to changes in the neuroretinal function before the onset of microvascular lesions in the human retina [19,20,21,22]. In addition, early thinning on the inner retina can be detected in type 2 diabetes by spectral- domain optical coherence tomography (SD-OCT) scans analysis, even before visible vascular changes are present, which supports the presence of a neurodegenerative process in diabetic patients [23].

**Figure 1:**
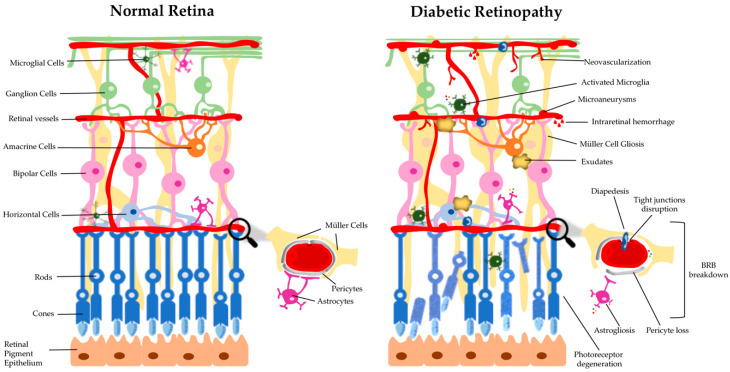
Schematic drawing of a cross-section of a healthy retina and a retina with diabetic retinopathy. The retina is a multilayered tissue composed by neuronal (photoreceptors, and horizontal, bipolar, amacrine and ganglion cells) and glial (astrocytes, Müller cells, and microglia) cells, that closely embed with three capillary plexuses. The blood-retinal barrier (BRB), that regulates the exchange of fluids between blood and retinal tissue, plays an important role in the maintenance of the retinal homeostasis. However, in diabetic retinopathy (DR) several retinal abnormalities appear, including microvascular changes (microaneurysms, intra-retinal hemorrhages, and neovascularization), the appearance of exudates, ganglion cell and photoreceptor degeneration, and glial dysfunction (astrogliosis, Müller cell gliosis, and activation of microglia). The reactivity of glial cells promotes the secretion of inflammatory cytokines, leukocyte adhesion, and diapedesis. These alterations lead to the breakdown of the BRB, characterized by pericyte loss and tight junctions’ disruption.

During the development and progression of DR several changes take place, which can be grouped into categories, such as physiological, rheological, hormonal, and biochemical, etc. [24]. It is well established that hyperglycemia contributes both to DR and other microvascular complications of diabetes, but it is not the single factor. There are several biochemical pathways that are involved in the onset and progression of DR. Moreover, no specific mechanism can be regarded as the main responsiblility for DR, rather a complex relationship between several pathways and factors may be important to the disease process. Functional and/or morphological changes are found in various retinal cell types, including endothelial cells, pericytes, microglial cells, ganglion cells, Müller cells, astrocytes, and microglia, in the diabetic retina, before clinical symptoms and diagnosis are attained (Figure 1) [25]. The development of the disease is believed to be due to a dysregulation of the neuroglial vascular unit. Nevertheless, this whole scenario is dynamic at different stages of the natural history of DR and varies from individual to individual [24,26]. In the first stage (preclinical retinopathy) there are significant biochemical and histological changes in the retinal vessels [27], which include increased permeability of the blood-retinal barrier (BRB), loss of perivascular cells, vascular basement membrane thickening with subsequent capillary occlusion, and neuronal and glial abnormalities. This is followed by a stage of morphostructural and pathophysiological changes, in which the progressive dysfunction of endothelial cells plays a crucial role, leading to worsening of previous changes and culminating in neovascularization (Figure 1) [28]. These changes are ophthalmoscopically visible and based on them, according to the multicenter Early Treatment DR Study (ETDRS), the DR may be classified as non-proliferative DR (NPDR) and proliferative DR (PDR) [29].

The presence of a few microaneurysms characterizes the mild NPDR while their presence associated with intraretinal hemorrhages or venous beading characterizes the moderated NPRD [30]. Progressively, retinopathy can be characterized by deposits of lipids in the retina (hard retinal exudates), small infarctions located in the retinal nerve fiber layer (cotton patches), collateral dilated capillary channels in areas of retinal ischemia (intraretinal microvascular abnormalities), and irregular dilation of retinal veins associated with significant retinal ischemia (venous beading). Then, the disease may further advance to a stage characterized by the development of new blood vessels (PDR) in the retina in response to oxygen deprivation through upregulation of angiogenic factors and, in some cases, development of fibrous tissue at the optic disc or near venules in the retina [31]. Advanced PDR is commonly associated with preretinal and vitreous hemorrhage as a result of the bleeding of new retinal blood vessels, and a traction on the macula due to fibrovascular tissue, which will then lead to a significant vision loss [24,32]. Notwithstanding, it is important to highlight an additional category of DR, which is characterized by leakage of the retinal vessels and thickening of the retina thus leading to what is called diabetic macular edema (DME). DME can be developed in all of the stages of retinopathy, although being more prevalent in the late stages of the disease, being the most common cause of vision loss in diabetic individuals [24,30,33].

## 3. The Role of Oxidative Stress and Inflammation in DR

The pathogenesis of DR is very complex and several mechanisms, such as hypoxia, oxidative stress, and inflammation are involved in the disease development and progression [30,34,35]. The retina is one of the most metabolically active tissues in the body, making it extremely sensitive to changes in oxygen levels [7]. In addition to the high content of polyunsaturated fatty acids (PUFAs), this tissue needs to produce energy (ATP) by consuming large amounts of glucose and oxygen, through the mitochondrial electron transport chain in the inner mitochondrial membrane. During this process, the electron transport chain can leak electrons directly onto molecular oxygen, generating free radicals/reactive oxygen species (ROS) [36]. Mitochondria are an important endogenous source of ROS in the retina. Although ROS play an important role in the immune response, excessive intracellular levels of ROS may damage cellular lipids, proteins, and nucleic acids. When ROS production exceeds cellular antioxidant capacity, ROS can thus contribute in a large scale to oxidative stress due to the limited mitochondrial reserve capacity, being particularly vulnerable to small changes in energy homeostasis [37]. Therefore, ROS can cause oxidative stress, damaging mitochondria [2], which results in a reduced efficiency of ATP production [36]. In the context of hyperglycemia, it has been shown that there is a dysregulation of mitochondrial biogenesis, leading to bioenergetics deficits [38]. Moreover, mitochondrial functional changes were detected in the retinas of diabetic rodents, before clinical manifestations of DR (pericyte loss and capillary degeneration) are present. Altogether, these data suggest that oxidative stress and mitochondrial dysfunction may play an important role in the onset and progression of DR.

The contribution of mitochondria to diabetes-induced oxidative stress is well established and, according to Brownlee [39], in his “unifying theory” of hyperglycemia-induced endothelial cell damage, ROS overproduction is the common upstream event that can stimulate the biochemical pathways which have a pathogenic role in DR: 1) The polyol (sorbitol) pathway, which can deplete the cytosolic nicotinamide adenine dinucleotide phosphate (NADPH), necessary to maintain glutathione (GSH), the main intracellular antioxidant, in its reduced state [24]; 2) the formation of advanced glycation end products (AGEs) intracellularly, which can induce cross-linking of proteins to promote vascular stiffness and enhance oxidative stress; 3) activation of different isoforms of protein kinase C (PKC) can induce changes in endothelial cell monolayer permeability (PKCα), cell proliferation (PKCβ) and increased ROS production, activation of the NF-kB, and platelet-derived growth factor (PDGF) survival signaling pathway (PKCδ) [40,41]; and 4) the hexosamine pathway, which decreases NADPH-dependent GSH production, is implicated in the apoptosis of endothelial cells and the limited proliferation of pericytes, as well as retinal neuronal apoptosis [42,43,44].

When intracellular antioxidant enzymes fail to remove efficiently ROS that are produced by retinal cells, excessive ROS can be accumulated within the cell cytoplasm, mitochondria, and nucleus. In the nucleus, ROS can cause DNA strand breaks [45,46].

Chronic hyperglycemia can trigger Müller cells and astrocytes reactivity, activating the transcription nuclear factor NF-κB [18]. Once activated, NF-κB is translocated into the nucleus, where it binds to nuclear DNA and promotes the expression of pro-inflammatory cytokines, such as interleukin (IL)-1β, IL-6, IL-8, interferon gamma (IFN-γ), and tumor necrosis factor-alpha (TNF-α). On the other hand, activation of the PI3K/Akt/mTOR signaling pathway mediates the secretion of inflammatory cytokines by ROS induced-hyperglycemia itself. Moreover, it has been proposed that Müller cells can directly initiate retinal inflammation in diabetes through stimulation of cluster of differentiation 40 (CD40) and indirectly signal to microglia to elicit inflammation [47].

Chronic hyperglycemia-induced vascular dysfunction is mediated through increased levels of intercellular adhesion molecule 1 (ICAM-1) in endothelial cells, that results in leukocyte adhesion to the endothelium, change of BRB permeability, and thrombus formation [48]. Increased levels of other inflammatory mediators have been also found in the vitreous of diabetic patients with retinopathy, such as the monocyte chemotactic protein 1(MCP-1), that is an important chemotactic factor for monocytes, and vascular endothelial growth factor (VEGF), an important mediator of angiogenesis and effector of permeability [49]. Cyclooxygenase-2 (COX-2) is another inflammatory agent that is increased in diabetic retinas and is released by activated inflammatory cells and glial cells, and may play an important role in the degeneration of retinal capillaries [50]. Under inflammation conditions, this enzyme increases the synthesis of prostaglandins, which stabilizes hypoxia-induced factor-1 (HIF-1), leading to VEGF expression and NF-kB activation. In parallel, mitochondrial ROS and oxidized mtDNA, when released into the cytosol, are recognized as damaged associated molecular patterns (DAMPs) by cytosolic pattern recognition receptors (PRRs), including toll-like receptors, namely TLR4 and TLR9. This recognition triggers cell death by different pathways: A NLR family pyrin domain containing 3 (NLRP3) inflammasome is formed, which leads to activation of caspase-1 and secretion of IL-1β and IL-18, leading to pyroptosis, a highly inflammatory form of programmed cell death [2,30,36]. Other types of cell death can occur, such as apoptosis and autophagy-dependent cell death. Apoptosis can be triggered by matrix metalloproteinases 2 and 9 (MMP-2 and MMP-9) that compromise the mitochondrial membrane potential [51,52]. Autophagy can lead to cell death by modulation of mTOR/AMPK, activating caspase 3 [53].

In summary, oxidative stress and low-grade inflammation associated with chronic hyperglycemia are considered to play a key role in the onset and progression of DR, being difficult to pinpoint exactly which of the mechanisms/pathways are most important in the pathogenesis of DR, rather the coexistence between them contribute to BRB breakdown and neovascularization [30]. Therefore, hyperglycemia leads to a series of successive triggered events, leading to neural, glial, and microvascular dysfunction, that culminate in DR [54].

## 4. Crosstalk between Endothelial Cells and Other Retinal Cells through Extracellular Vesicles (EVs)

**Figure 2:**
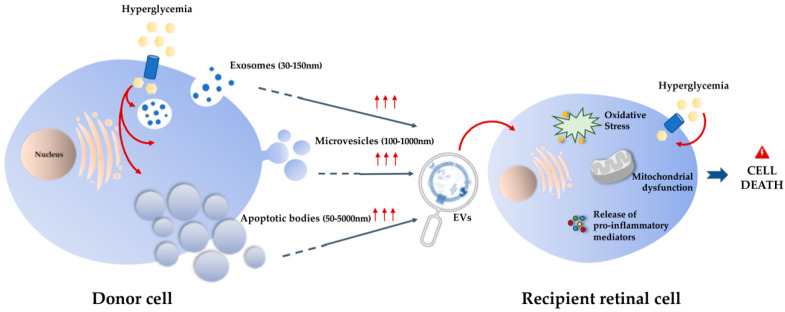
Extracellular vesicles (EVs) and their role in the pathogenesis of diabetic retinopathy (DR). EVs can be classified into three different types depending on their size and biogenesis—exosomes, microvesicles, and apoptotic bodies. Hyperglycemia leads to the increase of EVs released from donor cells. These EVs, derived from other retinal cells or from circulation, can interact with recipient/target retinal cells contributing, along with chronic hyperglycemia, to oxidative stress and release of pro-inflammatory mediators. These mechanisms may contribute to mitochondrial dysfunction leading to retinal cell death.

Intercellular communication is an essential component in all multicellular organisms, ensuring the exchange of information between cells in response to normal homeostatic processes or to possible pathological threats. One of the most important components involved in both short- and long-distance communication are the EVs, a heterogenous group of cell-derived membrane vesicles limited by a lipid bilayer that are secreted from almost all cell types and contain proteins, lipids, and nucleic acids, such as mRNAs and miRNAs [11,55]. Depending on their size and biogenesis pathway, EVs can be classified into exosomes, microvesicles, or apoptotic bodies. Exosomes are nano-sized vesicles of endocytic origin, with a diameter between 30–150 nm. This subtype of vesicles is originated through the inward budding of a multivesicular body (MVB) membrane that fuses with the cell membrane releasing the intraluminal vesicles (ILVs) to the extracellular space which generate the exosomes [56]. Microvesicles, also known as ectosomes or microparticles, are cell membrane-derived vesicles with a size range between 100–1000 nm. These vesicles are formed through the direct outward budding of the plasma membrane after vertical trafficking of a specific molecular cargo and consequent release of the microvesicles [57]. Apoptotic bodies are the largest subtype of EVs, with a wide range of sizes between 50–5000 nm, that are released also by outward budding of the cell membrane, exclusively during apoptotic cell death (Figure 2) [58].

All types of EVs normally reflect the phenotype of their parental cells, since different cells secrete different EVs with specific cargo molecules depending on their function. This suggests that EVs molecular profile can be used to detect not only their origin but also somehow the molecular content of the cell of origin. This feature makes these vesicles very interesting candidates as biomarkers and therapeutic drug delivery systems for a variety of chronic diseases, including cancer and degenerative diseases, such as DR [59]. A recent study characterized the EVs released by adult neural retina in culture and this allowed to determine the cellular origin of different types of EVs through the analysis of their molecular cargo (RNA and proteins) [60]. The authors were able to detect EVs derived from photoreceptors which contain proteins such as rhodopsin, the photo-responsive receptor of rod cells, and cadherin related family member 1 (Cdhr1), an adhesion protein that is normally present in the outer and inner segments of photoreceptors. They have also detected the presence of neuronal-specific nuclear protein (NeuN) in the isolated EVs, a marker for amacrine and retinal ganglion cells. Additionally, through their molecular content, they were able to conclude that the retinal-derived EVs are mainly related with processes such as phototransduction, synapse structure, RNA processing, and transcription regulation [60]. Furthermore, other studies have also demonstrated that EVs from distinct retinal cells, such as astrocytes and retinal pigment epithelial (RPE) cells present different protein profiles, which suffer alterations during pathophysiological processes [61,62]. Therefore, besides intercellular communication being essential to the maintenance of retinal function, a small alteration in this balance can lead to the appearance of several retinal diseases. Although EVs biology in the visual system is not extensively investigated, some studies have already described that EVs are closely involved in the progression of several retinal diseases, such as age-related macular degeneration (AMD) and glaucoma [63,64].

It is known that diabetes and its macro- and microvascular complications are associated to increased levels of EVs with distinct molecular profiles [65], presenting for instance procoagulant, proinflammatory, and proangiogenic properties [66]. We can speculate that in the diabetic eye, and in particular in the retina, a single alteration in the number of EVs is able to disturb the normal visual homeostasis (Figure 2). In fact, EVs biological effects in the eye were described to be concentration-dependent at their target site [67]. Concerning DR, the chronic low-grade inflammation and oxidative stress, which play an important role in diabetes progression, are closely related with the dysfunction of metabolic pathways in retinal endothelial cells. Early diabetes-related endothelial dysfunction can lead to a deregulation of intercellular communication between retinal endothelial cells, leukocytes, and neuroglial cells, which results in increased BRB permeability [30]. This deregulation of intercellular communication between retinal cells is closely related to the release of EVs with different profiles. For example, a report involving diabetic patients showed the presence of increased levels of photoreceptor-derived and microglial-derived microvesicles in the vitreous of patients with proliferative DR. Additionally, this study also demonstrated that these EVs were able to stimulate endothelial cell proliferation and neovascularization both in vitro and in vivo, confirming their different molecular cargo in the context of hyperglycemia [68]. Regarding DR progression, the intercellular communication between retinal endothelial cells and pericytes is critical for the vascular damage present in the eye of diabetic patients [14]. A recent study has highlighted the role of exosomes containing a specific circular RNA (circRNA) in the progression of DR. In that study, the authors demonstrated that circRNA cPWW2P2A, which is upregulated in pericytes during the disease, is transferred through exosomes to endothelial cells contributing to retinal vascular dysfunction [69]. In the same context, another study has demonstrated that under diabetic-like conditions, mesenchymal stem cells-derived EVs were able to cause pericyte detachment and endothelial cell proliferation, which may be mediated by MMP-2, with consequent BRB disruption [70]. In this study, the authors have also addressed the involvement of EVs-derived MMPs in the progression of DR. In fact, several MMPs, such as MMP-2, MMP-9, and MMP-14 have been described as being increased in the vitreous fluid and in the retina of both patients with DR and animal models of DR, contributing to vessel destabilization and consequent BRB breakdown [71].

Other important mediators involved in DR onset and progression appear to be closely related with EVs-mediated communication. In fact, TNF-α, C-reactive protein and thrombin, commonly increased in the eye of diabetic patients, can stimulate the formation of endothelial microvesicles in vitro [72,73,74]. As a consequence, the increased release of endothelial EVs can stimulate the production of ROS in the target cells, which may contribute to retinal vascular damage in the context of DR progression [75]. Nonetheless, in the early stages of the disease, EVs can also exhibit protective effects, preventing the rapid progression of the retinal damage. For example, in vitro and in vivo studies have demonstrated that EVs derived from microglial cells were able to inhibit hypoxia-induced photoreceptor apoptosis, thus preventing neovascularization and alleviating visual injury [76].

All these studies highlighted the importance of a tight regulated intercellular communication between retinal cells and the role of retinal-derived EVs in the progression of retinal disorders, namely DR.

## 5. Contribution of Plasma EVs to Microvascular Damage in DR

With diabetes being a chronic systemic disorder, circulating EVs have an important role in the progression of several complications associated with this disease. Increased levels of cytokines (namely TNF-α), angiogenic factors, RANTES (regulated on activation, normal T-cell expressed and secreted), and angiotensin-2 have been detected in the plasma EVs of diabetic patients, highlighting their role in diabetes progression [77]. Although some EVs can pass the BRB [59], they can also act pathologically in the BRB. During the development of DR, increased vascular permeability can also facilitate the accumulation of these circulating EVs in the eye, which may be crucial to the development of the most progressive forms of the disease. In this sense, several studies have been reporting the role of plasma EVs on the activation of inflammatory and oxidative mechanisms involved in the microvascular damage in DR (Figure 2) [78].

It is known that the complement activation, due to proinflammatory changes and impairment of complement regulatory proteins, has a main role in the vascular damage and DR progression [79,80]. A recent study from Huang et al. has proven the involvement of plasma EVs on complement activation in the retina, using a diabetic animal model [81]. Increased levels of plasma exosomes were associated with increased levels of IgG, which was present in the membrane of the vesicles, resulting in the activation of the classic complement pathway. Additionally, the lack of IgG-laden exosomes resulted in a decrease of complement activation and consequent reduction of retinal vascular damage, proving the involvement of the circulating EVs in the microvascular damage through complement activation [81]. In the same way, a more recent study from the same group has demonstrated that, using the same animal model and primary cultures of human retinal endothelial cells (hRECs), this mechanism, by which plasma EVs can lead to retinal vascular damage, occurs due to the deposition of membrane attack complex (MAC) and cytosolic damage [82]. In fact, increased levels of MAC on the eyes of diabetic patients have been already described, when compared to the eyes of non-diabetic patients [83]. In the end, the IgG-laden plasma exosomes may contribute to the microvascular damage in the context of DR activating the classic complement pathway which leads to MAC deposition and consequent endothelial damage.

Concerning plasma EVs, both platelet- and monocyte-derived microvesicles have been receiving a lot of attention in this field, since two studies from Ogata et al. have described increased levels of both types of EVs in diabetic patients with DR. These vesicles appear to be closely related with DR progression as their levels were increased in advanced stages of DR [84,85]. Specifically, platelet-derived vesicles appear to mediate hyperglycemia-induced retinal endothelial damage through the release of CXCL10 which is going to activate the TLR4 pathway. These platelet-derived EVs also induce the production of ROS and inhibit the activity of superoxide dismutase (SOD) which, in addition to TLR4 activation, leads to decreased levels of tight junction proteins, such as ZO-1 and occludin, and retinal endothelial injury, including BRB breakdown [86].

Other studies have correlated changes in plasma EVs molecular profile with DR progression. For example, a recent study has reported a correlation of RANTES and CCR5-positive microvesicles, both proangiogenic factors, with the progression of NPDR [87]. Furthermore, high glucose conditions are able to increase NADPH oxidase activity on endothelium-derived microparticles, which are also increased in diabetic patients, leading to increased levels of ROS and inflammatory mediators and consequent impaired endothelial function [88]. These circulating EVs may also contribute to early endothelial dysfunction by decreasing nitric oxide (NO) and prostacyclin activity, increasing macrophage and leukocyte infiltration [71]. Thus, EVs appear to be closely related with the endothelial dysfunction and microvascular damage in DR.

Some studies have described a procoagulant property of these EVs in the context of DR [89], suggesting that plasma EVs may play a role in the coagulation cascade during the progression of the disease. Moreover, Su et al. described that increased levels of phosphatidylserine (PS)-positive microvesicles in patients with non-proliferative and proliferative DR are related with an increased procoagulant activity, which may explain the role of EVs on microvascular complications in patients with DR [90].

Together, these studies highlight the role of plasma EVs in DR progression, which appear to be a great promise not only to use them as biomarkers but also as therapeutic targets for the treatment of DR in the future.

## 6. miRNA in DR: Role in Oxidative Stress and Inflammatory Signaling Pathways

**Figure 3:**
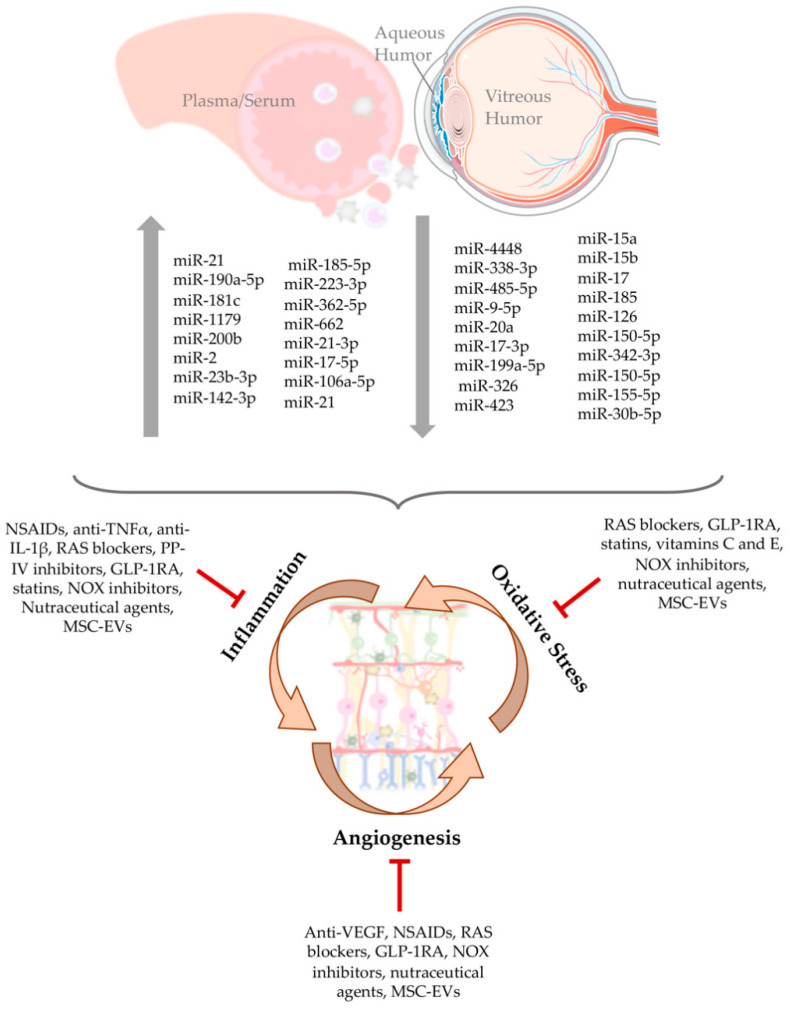
Alterations of miRNAs profile in DR and possible therapeutic interventions. The progression of DR is associated with the deregulation of miRNA expression, which leads to the increase (↑) or decrease (↓) of some miRNA involved in oxidative stress, inflammatory, and angiogenic processes. However, some therapeutic and nutraceutical strategies targeting inflammatory pathways, oxidative stress, and neovascularization, can be useful in preventing or arresting DR progression.

MicroRNA (miRNA) are a class of evolutionally conserved single-stranded RNA molecules. These are non-coding small molecules (19–25 nucleotides long) that are involved in the regulation of gene expression. In this way, they can affect nearly all aspects of cell physiology, such as cell growth, differentiation, metabolism, and apoptosis [12,91,92,93]. Dysregulation of miRNA expression has been extensively recognized to be linked to the development of many diseases, including DR [94,95,96]. In fact, several reports have found an association of miRNA with the risk to develop DR [97,98,99]. Changes in circulating/extracellular miRNA expression levels in biological fluids, such as plasma, serum, vitreous, and aqueous humor in diabetic patients with retinopathy have been reported (Figure 3) [100,101,102,103]. An important and challenging question is whether these biofluid miRNAs execute protective/deleterious effects in the retina or simply serve as (read-outs) biosignatures that can aid in diagnosing and monitoring DR.

Extracellular miRNA in biological fluids can be found associated with proteins or enwrapped with vesicles [104]. In the former, miRNAs can be coupled with proteins, such as Argonaute (AGO) [105,106,107], nucleophosmin 1 (NPM1) [108], and high-density lipoproteins (HDLs) [109,110], and be released into the extracellular milieu; the binding to proteins protect miRNA from degradation by RNases, increasing their stability in body fluids. In the latter, miRNA can be incorporated in three broad secretory vesicles, based upon their mode of biogenesis: Exosomes, shedding microvesicles, and apoptotic bodies [111], and then released to the extracellular environment.

Multiple studies focusing in the identification of circulating miRNA from biofluids that differentiate between diabetic patients with retinopathy and diabetic patients without retinopathy or healthy controls have been published in the last years. Some of these studies have shown remarkable predictive value for detecting DR. Wang et al., in a cohort study with a small sample size, found five miRNAs (miR-4448, miR-338-3p, miR-485-5p and miR-9-5p, and miR-190a-5p) differentially expressed in serum samples in DR and non-DR groups [112]. The first four miRNAs were downregulated and the remaining (miR-190a-5p) was upregulated in T2D patients with retinopathy. The five miRNAs regulate 55 target genes, with a substantial overlapping with sirtuins, which are known to play a significant role by influencing various pathological processes such as inflammation, oxidative stress, and angiogenesis [113].

In another study, the expression of two miRNA, miR-20b and miR-17-3p, using also a relatively small sample size, has been investigated [114]. MiR-20b and miR-17-3p belong to the miR-17 family and target the 3′ UTRs of genes encoding HIF-1α and VEGF-A. Therefore, they may play a role in angiogenesis in DR. While a significant decrease in serum miR-20b was found in diabetic patients compared to healthy subjects, a decrease in serum miR-20b and miR-17-3p was found in NPDR and PDR groups when compared with healthy subjects. A combined analysis of these noncoding RNAs with two regulators of retinal endothelial dysfunction, Homebox antisense intergenic RNA (HOTAIR) and metastasis-associated lung adenocarcinoma transcript 1 (MALAT1), was able to discriminate diabetic patients without retinopathy from healthy controls and NPDR and PDR from diabetic patients without retinopathy, suggesting that miR-20b and miR-17-3p may be used as noninvasive biomarkers for screening of DR and the early diagnosis of PDR [115].

A study conducted by Qing et al. validated three miRNAs (elevated levels of plasma miR-21, miR-181c, and miR-1179) in predicting PDR [98]. A rise in plasma miR-21 levels in patients with T2D and PDR has also been found in another study [102]. From a mechanistic point of view, miR-21 seems to be involved in tumor-induced angiogenesis through targeting PTEN, leading to the activation of AKT and ERK1/2 signaling pathways, and thereby enhancing HIF-1α and VEGF expression [116]. Moreover, it has been also described to protect endothelial cells against high glucose-induced cytotoxicity [117]. Therefore, miR-21 may be associated with angiogenesis in a diabetic microenvironment. Concerning miR-181c, its levels are elevated in high glucose conditions, but they are inhibited in endothelial cells exposed to hypoxia, suggesting that it plays a key role in the regulation of angiogenesis in ischemia and in diabetes [118].

The information available regarding the expression profiles of the miRNAs in the vitreous of eyes with DR is scarce, mainly because healthy individuals cannot be used as controls due to ethical considerations. For example, Gomaa et al. reported increased VEGF and miR-200b expression levels in the vitreous of diabetic patients with PDR compared to age- and sex-matched nondiabetic individuals (nondiabetic patients indicated for pars plana vitrectomy due to idiopathic macular holes). In addition to being present in the vitreous, miR-200b has been described in neuronal, glial, and vascular elements in the retina [119]. MiR-200b expression was previously reported in the diabetic retina of experimental animal models of diabetes, but there was no consensus among the results of these studies [119,120,121]. Murray et al. found an upregulation of miR-200b in the retina of Akita mouse at nine months of age [120]. In agreement, Kovas et al. also reported increased expression levels of this miRNA both in the retina and retinal endothelial cells from diabetic rats after three months upon diabetes induction with streptozotocin (STZ) [121]. However, McArthur et al. reported decreased miR-200b expression levels in the retinas of STZ-induced diabetic rats after one month of diabetes [119]. As VEGF is one of the direct targets of miR200b, the rise of VEGF expression in DR may be attributable to the downregulation of miR-200b. In addition to the direct inhibitory effect of miR-200b on VEGF expression in response to diabetes, it may also mediate such effect indirectly via p300, a histone acetylation and transcription coactivator in malignancies, affecting in this way the gene expression of multiple vasoactive genes [122]. A recent cohort study using vitreous samples from PDR patients has revealed that miR-20a-5p, miR-23b-3p, miR-142-3p, miR-185-5p, miR-223-3p, miR-362-5p, and miR-662 expression levels were significantly higher compared to controls (vitreous from non-diabetic patients with macular holes), whereas miR-199a-5p and miR-326 were significantly lower [123]. Interestingly, all six overexpressed miRNAs have been previously described as potentially targeting proteins that are increased in the vitreous during PDR, such as VEGF-A, angiopoietin 2, PDGF-B, and connective tissue growth factor (CTGF). These authors claimed that the observed increase in miRNA expression can play a role in the regulation of angiogenesis and wound healing processes in the context of PDR, and the interplay between miRNA and their targets may result in a variety of gene expression profiles [123].

Several other studies tried to give insight into the mechanistic pathways possibly affected in DR by analyzing altered serum miRNA levels. Recently, Tamir et al. have performed a study involving 47 T2D patients (10 without retinopathy, 22 with NPDR, and 15 with PDR) and 22 healthy subjects, to study the potential involvement of 16 candidate miRNAs that were previously described in the literature to be altered in the plasma/serum of diabetic patients (miR423, miR-486-3p, miR-320a-3p, miR-320b, miR-200b-3p, miR-221-3p, miR-146a-5p, miR-183-5p, miR-122-5p, miR126-5p, miR-30d, miR-93-5p, miR-21, miR-27b-3p, let7f-5p, and miR-16-2-3p) [99,119,124,125,126,127,128,129,130,131,132,133,134,135,136,137,138,139]. They detected decreased miR-423 levels in T2D with PDR compared to healthy controls. Moreover, the authors found a correlation between lowered miR-423 in diabetic patients and VEGF, and an inverse correlation between NO and eNOS expression, suggesting a crosstalk between miR-423 and VEGF signaling, affecting the eNOS function. A recent report has found that five miRNAs—miR-15a, miR-15b, miR-17, miR20a, and miR-185—are downregulated in the aqueous humor of NPDR patients with DME, which is a leading cause of legal blindness in patients with T2D. Most of the downregulated miRNAs were related to inflammation, oxidative stress, and angiogenesis. MiR-15a has been associated with a dual anti-inflammatory and anti-angiogenic action. It has been reported that human retinal endothelial cells cultured in high glucose conditions decrease the expression levels of miR-15a [140]. Moreover, this downregulation in vascular cells is associated to increased leukostasis in the retina (an indication of retinal inflammation), together with activation of pro-inflammatory signaling of IL-1β, TNF-α, and NF-κB [140]. It has been suggested that miR-15a inhibits angiogenesis by binding to VEGF-A or Tie2 angiopoietin receptor [141,142]. Reduced levels of TNF-α and suppressor of cytokine signaling 3 (SOCS3) induced by miR-15b have been reported to inhibit insulin resistance in retinal endothelial cells [143]. MiR-17-5p is known to regulate hypoxia-induced NLRP3 inflammasome activation [144], downregulating inflammatory mediators such as IL-1β and TNF-α, and negatively regulates TLR4 expression [145]. MiR-20a has been shown to ameliorate the altered markers of endothelial function and oxidative stress as well as mediators of inflammation in an animal model of diabetes [146]. MiR-185 is decreased in diabetic patients and mice [147]. It is also known that miR-185 inhibits angiogenesis through direct interaction with stromal interaction molecule 1 (STIM1) in human microvascular endothelial cells [148].

Lu et al. detected decreased miR-126 serum content in diabetic patients with NPDR compared to healthy controls [149]. Moreover, it has been demonstrated that insulin receptor substrate 1 (IRS-1) is a target gene of miR-126 in endothelial cells and retinal pericytes in an animal model of DR. Furthermore, the interaction between them downregulates the expression of PI3K/Akt pathway proteins and negatively influences the viability and invasion of endothelial cells and retinal pericytes isolated from a mice model of DR [150]. The PI3K/Akt pathway is crucial for a long-term upregulation of VEGF, which is an angiogenic factor involved in the pathogenesis of DR. Therefore, miR-126 and its target gene IRS-1 may be promising molecular targets for the prevention and treatment of DR. Although it has been claimed that miR-126 can be used as a biomarker for early diagnosis of PDR, no single biomarker is enough to give a clear predictive sign of DR, being necessary a combination of molecular biomarkers, as well as anatomical and functional biomarkers, to increase the specificity and diagnosis accuracy.

Although there are many reports showing differences in circulating miRNAs in diabetic patients with retinopathy, the information regarding the importance of miRNA shuttled by EVs in the onset and progression of DR is scarce. In a recent study, using plasma from T1D subjects with PDR, the miRNA profiling patterns in the circulating EVs were different compared with healthy controls [151]. From 11 miRNA differentially expressed in DR patients in comparison with healthy controls, four were found to be upregulated, namely miR-21-3p, miR-17-5p, miR-106a-5p, and miR-21. These miRNAs were previously correlated to angiogenesis, inflammation, diabetes, and response to ischemia [152,153,154,155,156]. Three downregulated miRNA (miR-150-5p, miR-342-3p, miR-155-5p) were described as anti-angiogenic and also found to be decreased in T1D or T2D patients [155,157,158,159]. Furthermore, Mazzeo et al. [151] have shown that EVs isolated from the plasma of diabetic patients with retinopathy were able to induce pericyte detachment, increased permeability of pericyte/endothelial cell bilayers, and capillary-like tubular structures formation, which are some of the early features of DR. More recently, the same authors have investigated the role of miR-150-5p, miR-21-3p, and miR-30b-5p in the regulation of microvessels homeostasis and angiogenesis. They found the involvement of those miRNAs in abnormal angiogenesis and hypoxia-induced retinal injury characteristic of the diabetic eye [160]. Altogether, these data suggest that miRNA shuttled in EVs appears to be involved in the onset of DR, and miR-150-5p, miR-21-3p, and miR-30b-5p extracted from circulating EVs were identified as putative prognostic biomarkers for DR. 

## 7. Therapeutic and Nutraceutical Agents with Anti-Inflammatory and Antioxidant Properties against DR

In clinical terms, the management of DR has been focused on intensive control of glycemia, as well as blood pressure and lipidemia, together with therapeutic interventions, such as photocoagulation with argon laser and immunotherapy through the intravitreal injection of anti-VEGF drugs [161]. DR progression and loss of sight can be prevented or delayed, but the existing retinal blood vessels damage and lost neuronal cell functions are typically irreversible, which is a major concern because those treatments are particularly directed to late-stage DR. In addition, there are relevant limitations associated with the discomfort caused to patients, the poor effectiveness in some individuals, the possibility of long-term side effects, as well as the economic cost [161]. Thus, it is crucial to develop novel and more efficient strategies to treat or retard progression of DR in early stages. In this regard, therapeutic and nutraceutical strategies targeting inflammation and oxidative stress have received renewed attention in recent years (Figure 3) [7,162,163]. In a small cohort, anti-VEGF therapy has been associated with a lower expression of miR-23b-3p in PDR as compared to untreated PDR patients, which suggests the involvement of a regulatory mechanism [123]. However, in this study it was not investigated whether differences exist between responders and non-responders to anti-VEGF therapy. 

Corticosteroids have the ability to reduce inflammation by genomic and non-genomic mechanisms [162,164]. Regarding the genomic, corticosteroid-binding globulin (CBG) protein transports corticosteroid molecules through the serum and allows the entry into the cytoplasm after crossing the cell membrane, where it binds to a glucocorticoid receptor thus admitting penetration into the cell nucleus. By interfering with a diversity of genes modulated by specific miRNAs, corticosteroids can influence the production of hundreds of proteins involved in inflammation and cell metabolism, which in the eye can contribute to anti-inflammatory properties and preservation of BRB. In addition, corticosteroids act directly by extracellular mechanisms linked with reduction of permeability and induction of vasoconstriction of the blood vessels as well as related with alleviation of cellular swelling and stimulation of adenosine production on Müller cells [162,164]. Furthermore, corticosteroids can stabilize blood–ocular barrier functions namely due to protection of tight junction integrity, which contributes to reduce serum leakage and the expression of extracellular MMP [165].

Currently, corticosteroids are viewed as a second-line therapy for DR patients that are poorly responsive to anti-VEGF therapy in cases of DME [166]. Triamcinolone acetonide, fluocinolone acetonide, and dexamethasone sodium phosphate have been successfully used as an intravitreal steroid treatment to reduce the frequency of anti-VEGF intravitreal injections, which is particularly useful in patients with contraindications for anti-VEGF therapy, such as those with coronary diseases [166]. Mounting evidence show that glucocorticoids exert anti-inflammatory activities by reducing the expression of adhesion molecules related to leukostasis (such as ICAM-1 and E-selectin), decreasing the release of other pro-inflammatory mediators (namely IL-6, NF-kB, TNF-α, and IFN-γ, etc.) and inhibiting the inflammatory cells (leucocytes, monocytes, macrophages) infiltration [167,168]. In vitro studies have shown that dexamethasone is able to reduce the secretion of exosomes containing pro-inflammatory miRNA-155 in RAW264.7 macrophages treated with lipopolysaccharide (LPS) [169]. Furthermore, glucocorticoids have been also associated with an angiostatic effect due to the inhibition of a variety of proangiogenesis mediators, namely VEGF, BFGF, and TGF-b [170,171]. Complementarily, in vitro and in vivo preclinical evidences have demonstrated that corticosteroids can also modulate vascular permeability by attenuating VEGF and SDF-1 pathways in different cell types and conditions [162], as well as exert neuroprotective effects on the retina [172].

Gathering evidences support a role for COX-2 in retinal inflammation, which opens up the possibility of using non-steroidal anti-inflammatory drugs (NSAIDs) in DR [50,173,174]. In diabetic animals, aspirin was able to prevent capillary cell apoptosis and vessel degeneration [50,175,176]. Regarding human data, the Dipyridamole Aspirin Microangiopathy of Diabetes (DAMAD) study reported beneficial effects of higher doses of aspirin (990 mg) in patients with early DR, in contrast to the poor results obtained in the advanced DR patients enrolled in the Early Treatment DR Study (ETDRS), using 650 mg of aspirin [177]. More recently, a prospective study showed beneficial effects of sulindac against DR development and progression [178]. Preclinical studies with specific COX-2 inhibitors have shown beneficial effects translated in reduced vascular leakage, capillary cell apoptosis, and vessel degeneration [179,180]. While the clinical use when administered systemically is discouraged due to increased risk of heart attack and stroke [164], the topical administration of a COX-2 inhibitor in preclinical studies was found to reduce DR symptoms similarly to systemic administration [174,179,180]. Topical use of NSAIDs in the eyes is overall of limited efficacy due to the reduced bioavailability and effect in the retina for the majority of these drugs, including bromfenac, nepafenac, and ketorolac [174]. To the best of our knowledge, no information is available in the literature concerning comparison of miRNA expression profiles in fluid samples between PDR patients treated with corticosteroids or NSAIDS and untreated PDR patients.

There are other therapeutic strategies under evaluation using inhibitors of proinflammatory molecules, such as the cytokines TNF-α and IL-1β, etc. Anti-TNF-α therapy has been mainly evaluated in preclinical studies and in a few cases of DME or PDR. A clinical study in a small number of patients with refractory DME were unable to present amelioration when treated with intravenous (IV) etanercept, a recombinant fusion protein having anti-TNF-α properties [181]. Intravenous therapy with infliximab, a monoclonal antibody directed against TNF-α, showed amelioration of visual acuity and reduction of macular thickness in DME patients non-responsive to laser photocoagulation [182]. However, other studies related with non-ophthalmic conditions, in which similar doses of infliximab were used, reported an increase in the incidence of serious adverse events [183,184]. Moreover, infliximab relieves BRB breakdown through the activation of the p38 MAPK pathway in a diabetic rodent model [185]. Regarding anti-IL-1β therapy with canakinumab (a selective IL-1β antibody), patients with proliferative DR presented stabilization (not regression) of retinal neovascularization [186].

Other drugs often used for other clinical conditions have been tested as anti-inflammatory agents against DR. Since the renin–angiotensin system (RAS) is involved in oxidative stress and AGEs formation, thus contributing to retinal inflammation in diabetes, RAS blockers have been evaluated in preclinical and clinical studies. Losartan and candesartan, which are angiotensin II type 1 receptor (AT1R) blockers, and enalapril, an angiotensin-converting enzyme (ACE) inhibitor, were able to promote beneficial effects against DR progression in animal models of diabetes due to the prevention of oxidative stress, inflammation, and vascular damage [187]. Similar benefits were observed in clinical trials [188], except in the DR Candesartan Trials (DIRECT), whose results showed reduced DR incidence but unaffected progression [189,190], recommending further research. MiR-152 has been pointed to be a regulator of the (Pro)renin receptor (PRR), a component of the RAS, in hREC. When hREC are exposed to high glucose conditions, PPR expression is induced via the inhibition of the miR-152, which is able to regulate the expression of VEGF, VEGFR-2, and TGFb1 [191].

Furthermore, dipeptidyl peptidase 4 (DPP-4) inhibitors (also known as gliptins), which are second-line oral anti-diabetic drugs, have been demonstrating anti-inflammatory properties and prevention of BRB breakdown in preclinical models of diabetes [192,193,194]. Similar benefits were obtained using glucagon-like peptide 1 receptor agonists (GLP1RAs) in experimental models, including protection against hyperglycemia-induced inflammation, oxidative stress, BRB breakdown, angiogenesis, and neurodegeneration, which seems to be at least in part mediated by the AKT pathway [195,196,197]. A recent report has described that liraglutide, a GLP1RA, has also vasoprotective effects in diabetic rats. Liraglutide was able to inhibit miR-93-5p, miR-181a-5p, and miR-34a-5p expression, and activate miR-26a-5p expression, which then stimulate the PI3K-Akt-Bcl2 activation pathway, thus inhibiting endothelial cell apoptosis [198].

Moreover, some lipid-lowering drugs, such as statins and fenofibrate, have been associated with protection against DR progression, which could be attributed to anti-inflammatory and antioxidant properties [199,200]. The promising results for some of these drugs in preclinical settings recommends further clinical evaluation in the perspective of possible repurposing to treat DR.

In preclinical studies, some vitamins (namely C and E) have been demonstrating antioxidant activity able to improve DR phenotype, particularly decreasing the development of acellular capillaries and the number of pericyte ghosts [201]. However, the clinical efficacy is contradictory. Some studies report beneficial effects whereas others mentioned a lack of a positive impact regardless of the antioxidant capacity [202]. Targeting specific promoters of oxidative stress could eventually be a more promising strategy. Several NAD(P)H-oxidase (NOX) inhibitors, including diphenyleneiodonium and apocynin, have been demonstrating preventive actions against DR progression, which might be due to reduction of ROS and VEGF levels, although NOX-independent effects have been also reported [203]. In preclinical studies, NOX blockers were able to reduce vascular leakage and neovascularization, as well as oxidative stress and inflammation, by mechanisms involving the prevention of NF-kB activation and CCL2 production [204,205,206]. It is known that in diabetes miRNAs can regulate the expression of ROS generating proteins, such as NOX, and antioxidant proteins, such as sirtuins or superoxide dismutase, influencing therefore the oxidative stress response [207]. However, the information available concerning the effect of NOX inhibitors on oxidative stress in DR is scarce. Further investigation is warranted in order to investigate whether changes of miRNA profiles may have an impact on regulating oxidative stress in the context of DR.

In recent years, much attention has been focused on the possibility that nutraceuticals agents can complement pharmacological therapy to prevent or delay the evolution of DR. Among the main candidates there are several natural molecules, including a variety of polyphenols (such as resveratrol, curcumin, quercetin, pterostilbene, epicatechin, epigallocatechin gallate, etc.) and anthocyanins, sesamin (a lignan isolated from sesame seeds and sesame oil), bromelain (a cysteine protease found in pineapple juice and stems), as well as alpha-lipoic acid (a vitamin-like chemical present in liver, kidney, and some vegetables), and lutein (a carotenoid present in green vegetables), etc. Several recently good reviews highlighted their strong anti-inflammatory and antioxidant properties, as well as their capacity to afford protection against hypoxia and angiogenesis, which have been associated with interference with adhesion, angiogenesis, and inflammation molecules/mediators (such as ICAM-1, VEGF, and TNF-a) and signaling (namely via NF-kB, NRF2-Keap1, and TLRs) [7,33,162,163]. These molecules could be an attractive nutraceutical alternative to the pharmacological approaches, but more clinical research is needed to complement the preclinical evidences. In addition, efficient delivery systems should be developed to overcome the well-known low bioavailability of some of them that still limits their efficacy.

As natural vehicles for the transfer of miRNAs, lipids, and proteins, EVs-based therapies have been recognized as having a number of potential applications for ocular diseases, namely DR. Particularly, stem cell (SC)-derived EVs are the most extensive explored since several studies have highlighted their positive therapeutic effects on immunomodulation and tissue remodeling without negative secondary effects [59]. Especially in the retina, pre-clinical studies have been using mesenchymal stem cells (MSC)-derived EVs to positively modulate injury responses. Intravitreal administration of MSC-derived exosomes are able to reduce retinal ischemia and neovascularization in a murine model of oxygen-induced retinopathy (OIR) [208] and reduce apoptosis and inflammatory responses through the reduction of MCP-1 in the retina of a mouse model of retinal laser injury [209]. In the context of DR, MSC-derived EVs also present protective effects in the retina of STZ-induced diabetic animals, being able to prevent retinal degeneration through the upregulation of miRNA-222 expression [210] and to reduce hyperglycemia-induced retinal inflammation, decreasing the levels of inflammatory markers, namely IL-1β, IL-18, and caspase-1 through miR-126 overexpression [211]. However, not only MSC-derived EVs present protective effects. In fact, Hajrasouliha et al. have shown that exosomes from retinal astrocytes cells were able to prevent retinal vessel leakage and inhibit neovascularization in a laser-induced choroidal neovascularization model [61]. These initial findings encourage further research and the development of novel EVs-based therapies for the treatment of DR.

## 8. Conclusions and Perspectives

The diagnosis of DR depends mainly on the detection of microvascular changes in the retina. Over the past decades, a significant progress has been made in the management of DR and a number of treatments are able to prevent, delay, or reduce vision loss. However, there is still no cure for DR. To tackle this challenge more effectively, early diagnosis is the most decisive factor.

Oxidative stress and low-grade inflammation play an important role in the pathogenesis of DR, but the exact molecular signaling pathways and key players involved are not completely elucidated yet. Evidence suggests that EVs can deliver their miRNA cargo to other cells, thus playing a role in cell-to-cell communication. As was highlighted in this manuscript, EVs and miRNAs might contribute to DR development through their important roles in inflammation, oxidative stress, and angiogenesis. However, the complete picture of miRNAs repertoire and their regulation in DR is highly complex and there are still many unknowns. As biomarkers, miRNAs do not yet allow to predict who will develop advanced forms of DR or to distinguish the stage of the disease. To take steps forward, some limitations associated with several studies must be overcome, such as small-cohorts, isolation of different subpopulations of vesicles or study groups. A better understanding of the role of EVs and changes in miRNA levels in DR could provide a more detailed characterization of the different stages of the disease. Future research approaches should be carefully charted to strengthen and confirm the current findings. As DR is a heterogenous and molecularly complex disease, miRNA-based therapeutics in combination with other anti-inflammatory and/or antioxidant therapeutic and nutraceutical agents could be an interesting opportunity for future exploration in the context of personalized combination therapy.
